# Molding of Plasmonic Resonances in Metallic Nanostructures: Dependence of the Non-Linear Electric Permittivity on System Size and Temperature

**DOI:** 10.3390/ma6114879

**Published:** 2013-10-25

**Authors:** Alessandro Alabastri, Salvatore Tuccio, Andrea Giugni, Andrea Toma, Carlo Liberale, Gobind Das, Francesco De Angelis, Enzo Di Fabrizio, Remo Proietti Zaccaria

**Affiliations:** 1 Istituto Italiano di Tecnologia, Via Morego 30, Genova 16163, Italy; E-Mails: salvatore.tuccio@iit.it (S.T.); andrea.giugni@iit.it (A.G.); andrea.toma@iit.it (A.T.); carlo.liberale@iit.it (C.L.); gobind.das@iit.it (G.D.); francesco.deangelis@iit.it (F.A.); 2 King Abdullah University of Science and Technology (KAUST), Physical Science and Engineering (PSE) Division, Biological and Environmental Science and Engineering (BESE) Division, Thuwal 23955-6900, Kingdom of Saudi Arabia; E-Mail: enzo.difabrizio@kaust.edu.sa; 3 Bio-Nanotechnology and Engineering for Medicine (BIONEM), Department of Experimental and Clinical Medicine, University of Magna Graecia Viale Europa, Germaneto, Catanzaro 88100, Italy

**Keywords:** photonics, plasmonics, thermoplasmonics, temperature dependence, non-linear optics, nanostructures

## Abstract

In this paper, we review the principal theoretical models through which the dielectric function of metals can be described. Starting from the Drude assumptions for intraband transitions, we show how this model can be improved by including interband absorption and temperature effect in the damping coefficients. Electronic scattering processes are described and included in the dielectric function, showing their role in determining plasmon lifetime at resonance. Relationships among permittivity, electric conductivity and refractive index are examined. Finally, a temperature dependent permittivity model is presented and is employed to predict temperature and non-linear field intensity dependence on commonly used plasmonic geometries, such as nanospheres.

## 1. Introduction

Plasmonics is the discipline describing the bridging between electromagnetic radiation and electronic oscillations. Recently, the research activity related to plasmonics has undergone a strong acceleration owing to the important role that it can play in different and important fields, such as nanomedicine [1,2,3,4,5], biosensing [6,7,8,9,10,11,12] nanophotonics [13,14,15,16,17,18,19,20,21,22,23,24,25,26,27,28], photovoltaic applications [29,30,31,32] or catalysis [33,34,35,36].

The fundamental quantity that is necessary for the designing and fabrication of reliable devices based on plasmonic resonances is the optical response of the system. Even though simple and intuitive models to generically describe the electric permittivity of a medium have been existing for a long time, practical applications require a precise knowledge of this quantity in different background conditions. Therefore, too simplistic frequency dependent models to describe the permittivity are not suitable for numerical calculations when the matching with experimental data is an issue. However, it is also unnecessary to employ very complex theoretical models, in fact quite often environment conditions simply do not require them: a more refined model is usually described with more parameters, which might be difficult and/or time expensive to determine. By collecting the efforts spent in the modeling of the electrical constants of noble metals, in this review we present how a theoretical model for the gold electric permittivity (but analogous considerations can be done for different kinds of metals) can, step-by-step, be gradually improved by including external parameters, which affect its value and thus the set of possible applications. We shall begin from the simple free electron Drude model, which accounts for intra-band transitions and we shall enrich it by progressively adding inter-band transitions (Lorentz model), size and shape dependence, temperature and other damping effects such as radiative relaxation and electron-surface scattering.

Particular attention will be spent on heating processes, since the merger between plasmonics and bio/medical applications relies on a full comprehension of how much and how fast plasmonic resonances might increase the temperature of biological tissues [37]. In particular, this aspect becomes crucial for cancer treatment by means of metallic nanoparticles where the process is specifically based on cell hyperthermia [4,5,38,39,40,41].

Our aim is to provide a framework to show how the different models can improve the valuation of the optical response in different work conditions highlighting shape/size and temperature effects.

## 2. Modeling the Optical Response of Metals: From Drude Model to Drude-Lorentz Model at Room Temperature

Describing how electromagnetic waves interact with matter is a fundamental step towards the knowledge of the physical processes happening in nature. The ability to model this interaction offers the possibility to quantify and engineer the involved physical parameters, leading to the design and the fabrication of advanced nanostructured optical devices.

Depending on the characteristics of the considered physical system (dimension, materials, timescale, *etc.*), there are different ways to model the ongoing optical phenomena. For instance, if light wavelength is much smaller of the system dimensions and only non-absorbing dielectrics or mirrors are involved, ray optics is able to precisely predict the light behavior. In this case, Snell’s law and refraction indexes of the materials forming the system are enough to obtain reliable results. In fact, the materials can be assumed homogeneous with no losses, essentially decoupling the electromagnetic properties of light from its propagation.

Historically, the efforts to properly describe the interaction of light with solids are backed to the beginning of 20th century when Drude proposed a relatively simple and intuitive transport model for electrons which could classically express the dispersive behavior of metals [42,43]. Actually, the Drude model (later modified by Lorentz) is at the base of all approaches which are currently used to describe the electric permittivity, therefore we shall start from its description and we shall see how it can be improved and tailored depending on applications and needs.

### 2.1. The Drude Model

A simple way to describe the interaction between an electromagnetic field and a generic material is to express the motion of its electrons through a forced, damped harmonic oscillator [44]. We can describe the system considering the displacement of an electric charge due to the force impressed by the incoming electric field:

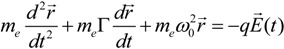
(1)


Regarding the left side, the first term expresses the acceleration of the charges induced by the electric field, the second one describes the damping factor due to electrons scattering where Γ is the damping coefficient, while the third term accounts for the restoring forces with a characteristic frequency *ω*_0_, typical of a harmonic oscillator. At the right side of the equation the driving term, *i.e.*, the force (depending on the electric field *E*) acting on each electron with charge *–q* *<* *0*. In Section 4 we will expand Γ and we will show its fundamental role when the temperature dependence is considered.

Since we are dealing with metals, we can temporary assume a free electrons behavior in the conduction band, hence we can neglect the restoring term (*ω*_0_ → 0). This approximation leads to the so called Drude model, namely only intra-band transitions will be considered. In this way, we can recast the damped oscillator equation in a more convenient way. In fact, by considering the oscillatory time dependence *e^−iωt^* of the electric field (which can be extended to the displacement quantity *r*), and by applying the Fourier transform properties twice we obtain (the vectorial form was dropped for simplicity):

*r*(−*ω^2^m_e_* − *iωm_e_*Γ) = −*qE* (2)

By defining the electric polarization density *P* as the magnitude of the induced electric dipole moments, we obtain the following set of equations [43,45].


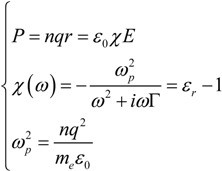
(3)
where *ω_p_* is termed bulk plasma frequency, *ε*_0_ is the vacuum electric permittivity and *χ* is the electric susceptibility. Interestingly, the polarization density is usually defined to describe dielectric properties of materials however, within metals, we could argue that the polarization should diverge since bulk electrons are not bound to ions. However, we have to remember that these equations assume alternating electric field with no drift currents. Therefore, *r* can be seen as the oscillating displacement of electrons from the initial position. Eventually we can extrapolate the electric permittivity as:

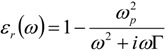
(4)


We recall that only intra-band transitions have been considered. In order to understand the importance of this parameter we have to remind that the electric permittivity rules the electromagnetic behavior during the wave propagation. In fact, the wave equation calculated from Maxwell’s laws explicitly sets it as the leading term which translates the media physical properties into optical phenomena:

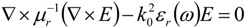
(5)


Here *μ_r_* is the magnetic permeability and *k*_0_ the vacuum wave vector. It is clear that being able to quantify and modify the electric permittivity means controlling the optical response of the medium.

It is worth noticing that we could have reached the same result considering the metallic behavior through a frequency dependent electric conductivity. Beginning from the same Newton Equation (1), and deriving just one time, we can describe the same system in terms of oscillating charge velocity instead of displacement, that is:

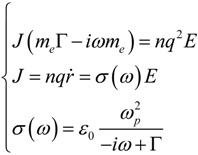
(6)
where *J* represents the current density (electric current per unit area). We see that the electric conductivity *σ* represents the proportionality term between the current density and the electric field amplitude. To be noticed that the DC conductivity can be promptly recovered from this expression:

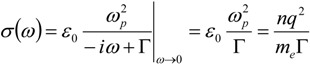
(7)


Comparing this set of equations with Equation (3), we can find a direct relation between refractive index, permittivity, conductivity and susceptibility, namely:

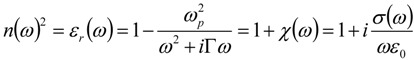
(8)


With this formalism, we can treat dielectric and metallic media in the same way. All the information is included either in the electric permittivity or in the conductivity.

#### Relations between Refractive Index, Permittivity and Conductivity

**Figure 1:**
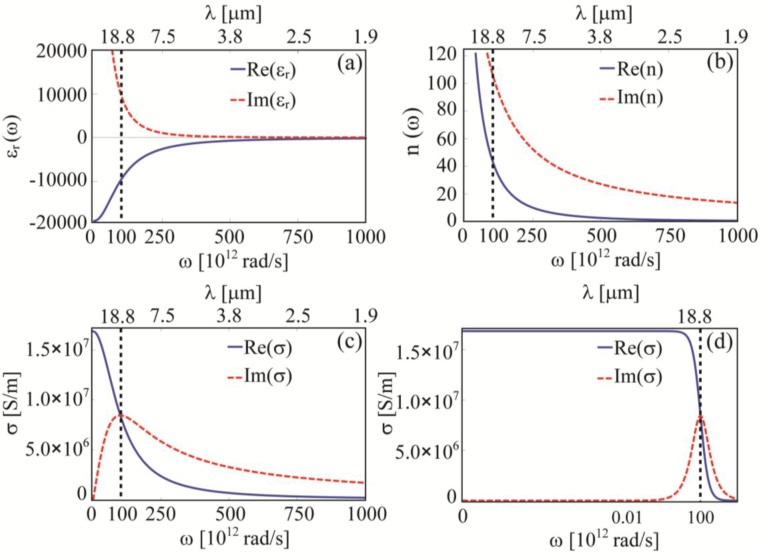
(**a**) Real (blue, full) and imaginary part (red, dashed) of gold relative permittivity based on Equation (8); (**b**) Real (blue, full) and imaginary part (red, dashed) of gold refractive index based on Equation (8); (**c**) Real (blue, full) and imaginary part (red, dashed) of gold conductivity based on Equation (7); the Γ parameter is highlighted; (**d**) Real (blue, full) and imaginary part (red, dashed) of gold conductivity based on Equation (7) in logarithmic scale; the Γ parameter is highlighted.

Considering the importance of the optical constants for electromagnetic analysis, it can be very useful to understand the reciprocal trends and the function of these structural parameters depending on the frequency. For instance, the quantities Γ and *ω_p_* for gold at room temperature [43] provide the behavior shown in Figure 1. In this case Γ = 1 × 10^14^ [rad/s] and *ω_p_* = 13.8 × 10^15^ [rad/s]. We notice that while *n* and *ε* (from now on with *ε* we shall mean the relative permittivity *ε_r_*) are monotonous over the considered frequency range, the real part of the conductivity *σ* drops at a certain frequency around Γ. In fact, at frequency above Γ, the frequency of the incident EM wave matches the electrons scattering process rate and this drastically reduces current density in the medium (*i*.*e*., the medium loses its metallic characteristics becoming dielectric-like). Even though the damping factor Γ cannot be freely tuned, it is important to understand its physical role and how it affects the media constitutive parameters. Therefore, it is interesting to notice that the role of Γ cannot be directly understood by looking at the *n* or *ε* plots. In particular, from *σ* plots (see Equation (7)), we see that the damping parameter Γ sets, at the same time, both the DC or low-frequency conductivity and the position of the pole of *σ*(*ω*). We recall that *σ*(*ω*) represents the proportionality term between *J* and *E* (see Equation (6)).

**Figure 2:**
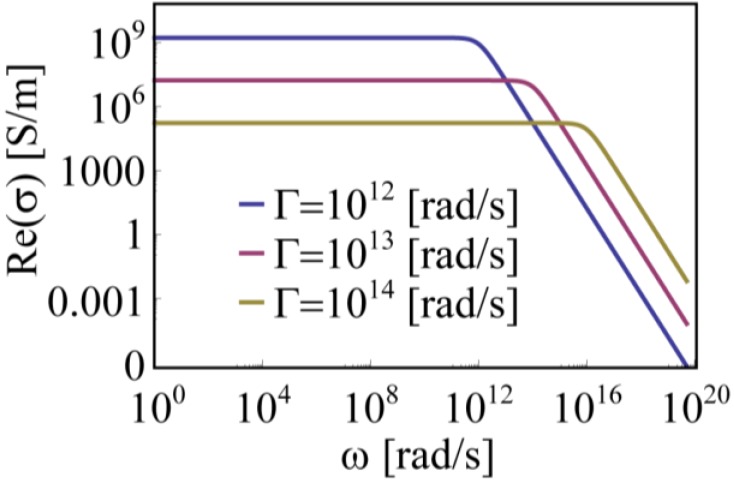
Real part of the conductivity based on Equation (7) for different values of Γ. Both axes are in logarithmic scale.

**Figure 3:**
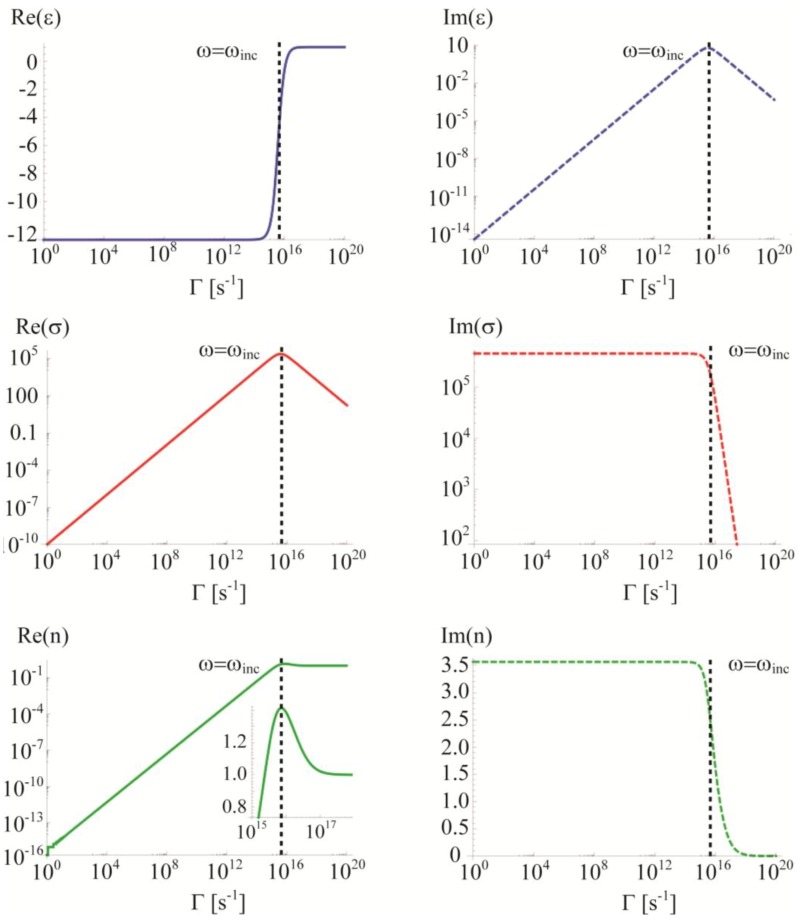
Real and imaginary parts of permittivity (*ε*), conductivity (*σ*) and refractive index (*n*) over a wide range of values of the damping factor at the frequency *ω_inc_* = 3.73 × 10^15^.

The logarithmic plot in Figure 2 reports, in a wide range of frequency, the shape of Re(*σ*) for three different values of Γ. It can be observed that the frequency at which Re(*σ*) drops can be blue shifted only by diminishing its low frequency (DC) value. We also notice that the Perfect Electric Conductor (PEC) condition (infinite Re(*σ*) in AC regime) cannot be recovered within this model by setting Γ = 0 as one may think by looking at Figure 2. In fact, in Figure 3, we report the behaviors of *ε*, *σ* and *n* over a wide range of Γ values for a given frequency, *ω_inc_* = 3.73 × 10^15^. From these plots we can extrapolate some important information. First, we see from Equation (4) that Γ = 0 leads to Im(*ε*) = 0 which represents a pure scatterer. Indeed, as shown by Equation (7), for Γ = 0 the conductivity *σ* is purely imaginary (Re(*σ*) = 0) since its real part would become infinite but over a *vanishing* spectrum. Therefore, the system does not absorb (it does not heat up). In the ideal case of a metallic nanoparticle (NP) with Γ = 0, the incident light would be completely radiatively re-emitted (scattered) without any dissipation. In this case the only energy leaking channel from the system would be radiation. On the other hand, Γ → ∞ leads again to Im(*ε*) = 0 (and to Re(*ε*) = 1, see Equations (8) and (9)), but now both the real and the imaginary parts of conductivity *σ* go to zero (see Equation (6)). Therefore, similarly to the Γ = 0 case, the dissipated power would be zero since it can be obtained by the Joule heating expression: *P_Joule_ = **J*** × ***E*** where ***E*** is the electric field vector moving the charges and ***J*** represents the current vector induced in the medium; the latter is zero if the conductivity vanishes (***J*** = Re(*σ*) ×***E***). Furthermore, considering that very high values of Γ imply Re(*ε*) = 1 and Im(*ε*) = 0, we can infer that this condition leads to lossless transparent medium. The behavior turning point is fixed by the operative frequency *ω_inc_* *=* 3.73 × 10^15^ as suggested by the plots in Figure 3.

In the article by Luk’yanchuk *et al.* [46] it was shown that, counter-intuitively, near the localized plasmon resonance the *maximal absorption* (dissipation) is reached for a *specific value of* Γ. At this point, it can be useful to underline the relationship between permittivity and conductivity:

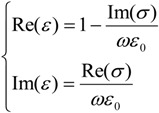
(9)


In fact, in order to obtain an elevated dissipated power, large values of **J** and **E** are needed within the metallic medium. The former is maximized by Im(*ε*) or Re(*σ*), the latter by the relation between Re(*ε*) and the structure geometry. For example, regarding a metallic nanoparticle, we can obtain a high electric field near its localized plasmon resonance [44] and the frequency at which the resonance occurs is mainly set by the particle geometry and the value of Re(*ε*) [47]. Obviously and importantly, this requests on Re(*ε*) will affect the value of Γ. At resonance, the incident power gets funneled in the particle due to diffraction [46] and its effective cross section exceeds its geometrical one [7,48]. Thus this condition, leading to a large ***E***, needs to match with the requirement of a large induced current density ***J*** (namely Re(*σ*)). From previous plots, it is clear that, depending on the chosen frequency, an optimal value of Γ exists for maximizing Re(*σ*): given a certain frequency *ω_inc_*, the maximum conductivity can be found by setting Γ*_σ-max_* = *ω_inc_* (this relation is easily demonstrated by calculating the dependence of Re(*σ*) on Γ, as it can be done starting from Equation (7)). Hence, by considering the double request on Γ originating from the maximization of both ***E*** and ***J***, it might occur that the optimal damping value Γ*_opt_* leading to the maximum dissipation can be different from Γ*_σ-max_.*

**Figure 4:**
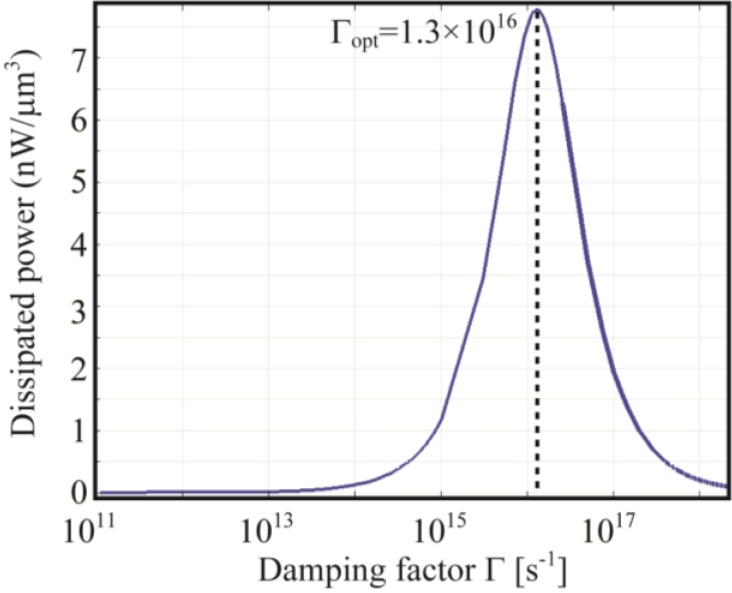
Dissipated power for a 10 nm radius Au NP in air over a wide range of Γ values at a given incident frequency: *ω_inc_* = 3.73 × 10^15^. The optimal value Γ*_opt_* maximizing the dissipated power is shown.

As an example, in Figure 4 the dissipated power is reported over a wide range of Γ values, for a gold nanoparticle in air when the frequency of the incident radiation is *ω_inc_* *=* 3.73 × 10^15^. The NP permittivity has been modeled according to Equation (8). The figure clearly shows that the *best absorber* condition in terms of dissipated power is reached for a precise value of Γ and that Γ*_opt_* is indeed different from *ω_inc_*.

Therefore, we understand that Γ plays a crucial role in the metallic systems and in the next chapters we will show its dependence on temperature and how it can be linked to the plasmonic resonance lifetime.

Even though it was possible to determine the conditions on the damping Γ to maximize the dissipated power, we need to consider more ingredients in the model of the permittivity in order to properly describe real systems. To do so, we need to consider also the interband transitions which happen to be crucial in red-shifting the localized plasmon resonances for metallic NPs [49,50]. In fact it has been shown experimentally [51] that gold NPs in solution (*n_solution_* ~ 1.5) resonate around 540 nm even though the Mie theory, based on the Drude model previously introduced [44], predicts a resonance at $\omega_{p}/\sqrt{3}$, *i.e.*, ~237 nm. Once again, we understand the importance of defining a correct modeling approach in order to obtain realistic results.

In the next section, we will show how the dielectric function can be properly modified in order to retrieve some fundamental experimental results.

### 2.2. Interband Transitions: The Lorentz Model

**Figure 5:**
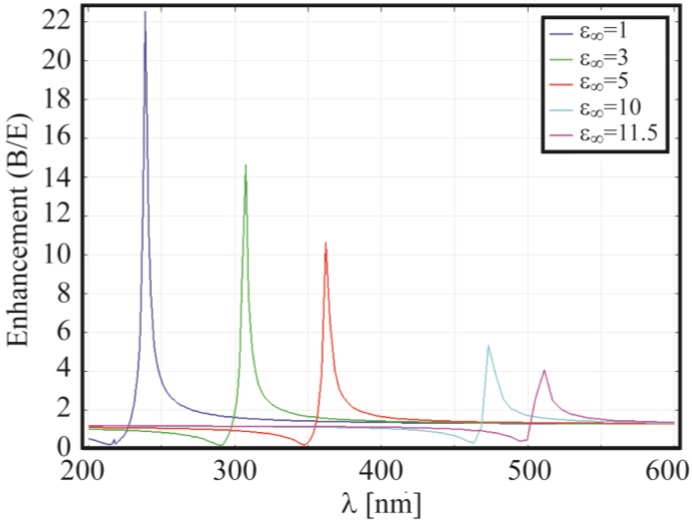
Electric field enhancement calculated at 1 nm from a gold nanoparticle. The particle radius is 5 nm and it is surrounded by air. Different values of *ε*_∞_ have been considered.

It has been mentioned that the combination of a particular geometry and a precise value of Re(*ε*) can trigger a resonance in a nanostructure. For a spherical NP in quasistatic approximation [52] the resonance is induced when Re(*ε*) = −2, the so called Froelich condition [44]. In fact, it can be shown that the polarizability *α* of a metallic sphere, under these assumptions, can be calculated as:

(10) $$\{\begin{array}{c} \alpha \propto \frac{4\text{π}\varepsilon_{0}\varepsilon_{d}B}{E}R^{3}=4\pi \varepsilon_{0}\varepsilon_{d}R^{3}\frac{\varepsilon -\varepsilon_{d}}{\varepsilon +2\varepsilon_{d}}\\ \varepsilon =\varepsilon^{\prime }+i\varepsilon^{\prime \prime } \end{array}$$

where *R* is the NP radius, *E* and *B* are is the amplitudes of the incident field and the field calculated outside the sphere, *ε_d_* and *ε* the permittivity of the embedding medium and the metal, respectively. When *ε'* ~ −2*ε_d_* the polarizability is maximized, and since *α* is proportional to *B/E*, which is related to the near field enhancement, it represents also the condition for the localized plasmon resonance. However, considering that the experimental resonance appears at longer wavelengths than the theoretical one found by using the Drude permittivity of Equation (4) with Γ = 0 (≈237 nm for gold), it is clear the necessity of theoretically modifying *ε* to properly red-shift the peak. In particular, in 1977 Beach and Christy [49] and later, in 1981, Parkins *et al.* [53], reported a significant effect on Re(*ε*) due to the presence of interband transitions. To approximate this effect they introduced into the Drude model a real positive term δ*ε* such that experimental results were recovered. In particular, considering that the general expression (Γ = 0) for the permittivity is given by *ε* = *ε*_∞_ − *ω_p_*^2^/*ω*^2^, the authors considered the possibility of substituting *ε*_∞_ with the quantity *ε*_∞_ = 1 +δ*ε* namely *ε* = (1 + δ*ε*−*ω_p_*^2^/*ω*^2^). In Figure 5 is plotted the field enhancement on a 5 nm radius NP for different values of *ε*_∞_.

The modification of the *ε*_∞_ parameter clearly tunes the resonance on a wide frequency range therefore it allows the matching between theoretical and experimental results. The actual *ε*_∞_ can be found by fitting the experimental data. For instance, in [54] it was set *ε*_∞_ = 11.5 for λ > 516 nm.

A more elegant and physical way to modify the dielectric function *ε*_m_ is to add the Lorentzian terms into the Drude expression in order to replicate the interband absorptions (Drude-Lorentz model). In 1998 Rakic *et al.* [50] combined several oscillators to reproduce the absorption peaks due to interband transitions up to 5 eV:

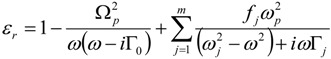
(11)


**Figure 6:**
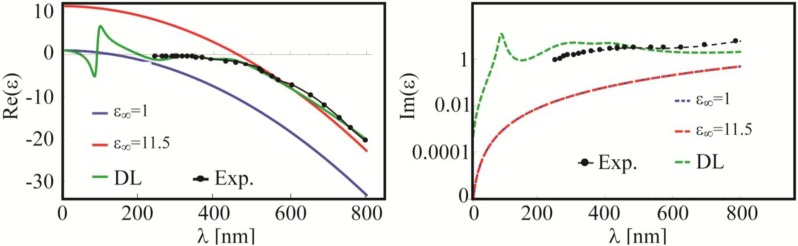
Real and imaginary part of gold permittivity. Comparison between Drude model with *ε*_∞_ = 1 (blue lines), *ε*_∞_ = 11.5 (red lines), Drude-Lorentz (DL, green lines) as proposed by Rakic in [50] and experimental data (black lines) from [55].

Here *m* stands for the total number of the oscillators, each with lifetime Γ*_j_*, strength *f_j_* and frequency $\omega_{j}{\text{Ω}}_{p}=\sqrt{f_{0}}\omega_{p}$ is the modified plasma frequency including the oscillator strength *f*_0_. In Figure 6 the real and the imaginary parts of the permittivity are plotted by using this last approach, and they are compared with the Drude model.

**Figure 7:**
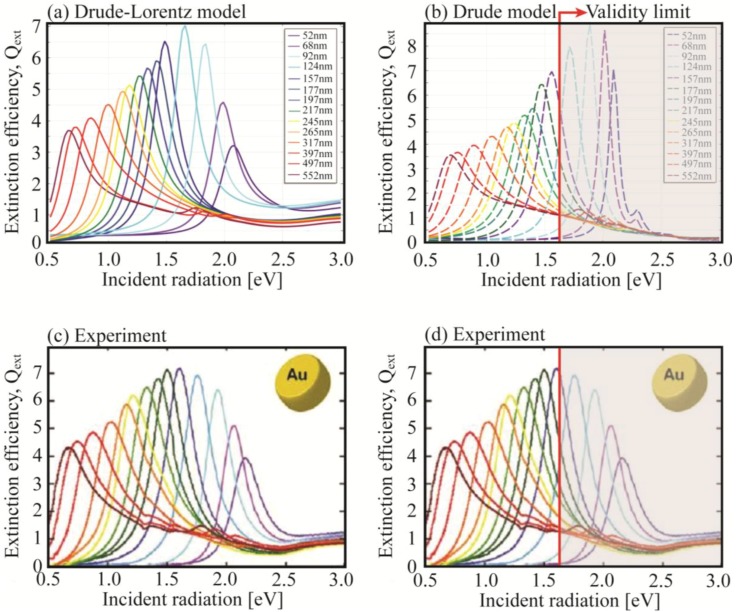
Extinction efficiency of Au nanodisks of different diameters at a fixed height H = 20 nm. (**a**) Drude-Lorentz model (see Equation (10)); (**b**) Drude model with *ε*_∞_ = 11.5. The red line highlights the validity range of the Drude model (**c**,**d**) Experimental measurements from [64]. The red line highlights the validity range of the Drude model. Reprinted with permission from [64]. Copyright 2011 American Chemical Society.

Regarding the real part, we see how the DL model and the Drude model with *ε*_∞_ = 11.5 are quite similar to each other in most of the visible region (>500 nm) while the Drude model with *ε*_∞_ = 1 is shifted to lower wavelengths. Whereas the DL model holds all over the spectrum [50], the Drude model with *ε*_∞_ = 11.5 was obtained by fitting the data below the interband transition frequencies (>516 nm). For higher frequencies, such as the UV region, this model is not capable of reproducing the experimental data. Turning our attention toward the imaginary part of the permittivity, we have to recall that the DL model is based on a set of absorption resonances defined by the typical Lorentzian shape. On the other hand, both the standard (*ε*_∞_ = 1) and modified (*ε*_∞_ > 1) Drude models overlap each other because *ε*_∞_ affects only the real part of the permittivity. This is an important point: in fact, if only the resonance positions need to be found, then Drude model with *ε***_∞_** = 11.5 works fine for energies below 2.4 eV (516 nm). However, as previously explained, if the dissipated power and the absorption cross section must be evaluated, the models will produce anomalous results. In Figure 7, experimental measurements of extinction efficiencies are compared by using DL and Drude (*ε*_∞_ = 11.5) models.

From the plots, we can see how the DL permittivity gives a nice matching (both for the peaks position and the line widths) with the experimental measurements for all the diameters of the nanodisks. Instead, when the Drude model with *ε*_∞_ = 11.5 is employed, we observe a sufficient agreement only below 1.6 eV. At higher energies (shorter disks diameters), while the peaks positions are correct, both extinction values and line-width are not reliable when compared to experiments.

Finally, it is worthy noticing the existence of at least two more analytical methods for describing the optical properties of materials. In 1992, Brendel and Bormann [56] replaced the Lorentz oscillator with a Gaussian-like term to describe the interband contribution. This lead to a slightly better fitting of the experimental data when compared to the Drude-Lorentz model. Similarly, in 2006 Etchegoin and co-workers [57,58] modeled the gold permittivity based on critical point analysis of Au interband transitions. Along with this method, they could fairly reproduce the Johnson and Christy Au experimental data [59] with a relatively small number of fitting parameters. The critical point analysis [60,61,62] employs a family of analytical models to describe the joint density of states in the vicinity of interband transitions [63]. When modeling metals permittivity, in order to describe the asymmetric line shape of interband absorptions, this approach requires fewer terms if compared to the use of multiple Lorentzian oscillators [57]. However, it must be pointed out that the damping coefficient Γ_0_, comprised in the Drude term of Equation (10), is not affected by the method employed to describe the interband terms. In fact, this damping factor is associated with the scattering mechanisms of electrons within the conduction band and it is thus independent of the extra terms related to valence band electrons.

## 3. Heating Processes in Plasmonic Nanostructures

The temperature dependence of the damping factor is an important aspect because plasmonic nanostructures tend to heat up when irradiated by an external light source: dissipated power due to the Joule effect increases the temperature of both the metallic particles and the surrounding media. As already mentioned, the fraction of the incident power which can be converted to heat, may depend on several factors. Since resonances are dependent on both geometry and permittivity we have many possibilities to tune the enhancement spectrum shape of metallic nanoparticles. During the last decade, photothermal effects in plasmonic nanoparticles have been intensively studied both from a theoretical and analytical point of view. In 2006, Govorov and coworkers [65] studied NPs ensembles as heaters capable to melt a surrounding ice or a polymer matrix. They also reviewed the plasmonic heating process and characterized the heat generated in gold NPs ensembles in [66]. In 2010 Baffou and coworkers [67] the related heat sources to the optical hot spots inside plasmonic structures showing that they do not usually match one another. Once again, this finding can be explained by the different conditions leading to maximum current J and field enhancement E. More complex geometries have also been investigated. Rodriguez-Oliveros and Sanchez-Gil [68] numerically studied gold nanostars as optical heaters finding peculiar thermoplasmonic properties such as LSPR tunability and high absorption/scattering ratios depending on the geometrical symmetries and on the number of tips of the structure. Juen Tan and Gramotnev [69] investigated the heating mechanisms in metal wedges and they proposed a model to separate plasmon propagation from heat generation if the dependence of the permittivity on temperature can be neglected. In addition, tip-shaped structures have been analyzed and the relationships between high field enhancement and temperature increase have been pointed out [70,71,72]. In 2002 Sonnichsen *et al.* [73] showed a drastic reduction in the plasmon damping of gold nanorods compared to spherical NPs. This is due to the fact that when the plasmonic resonance is below the gold interband transitions energies, the damping factor due to interband recombination is very small and the plasmon lifetime grows while the resonance line width shrinks. The shape of a nanostructure can indeed influence its resonant behavior in two ways: (i) through the relationship between its geometry and the real part of the permittivity and (ii) through the effective length L_eff_ which determines the damping factor due to the electron-surface scattering (see Equation (16)). The latter influences the plasmon lifetime, by increasing or decreasing the scattering rate depending on the *V/S* ratio of the particle. However, the shape implicitly determines the resonant condition and thus indirectly contributes to the entity of the damping factors. In 2000 Link and El-Sayed [74] extensively studied the connection between shape and size of nanoparticles with the corresponding radiative and non radiative scattering rates. In 2008 Pelton *et al.* [75] reviewed plasmonic properties associated with metal-nanoparticles pointing out how the geometry impacts on NPs and nanorods resonance positions, highlighting the limits of quasi-static approximation utilized in analytical calculations. Later in 2009 Baffou and coworkers [76,77] studied the influence of the morphology on the resonances and the heat generation of plasmonic nanostructures employing the Green’ dyadic method. They found that for a spherical NP only the outer part of the particle participates in the heating generation process, therefore more elongated structures, such as nanorods, show a diminished shielding effect and the heating is more efficient. In fact they demonstrated that, considering the same structure volume, thinner particles and those presenting corners or sharp edges are more effective in producing heat. Other studies on this subject can also be found in [71,78,79,80,81] where the effect of heating is also considered in relation with the re-shaping of a particle close to its melting temperature. In 2012, Chen *et al.* [82] studied photothermal effects in gold nanospheres finding structures entering a pre-melting regime at 519 K and changing sensitively shape around 795 K, much lower than bulk gold melting point (1337 K). Photothermal interaction between nanospheres has also been proposed as a plasmon ruler by Zhang *et al.* in [83]; the effect is based on the sensitivity of the absorption power on the distance between two NPs placed on a substrate. Being able to measure the absorption differences, can lead to nanometric gaps characterizations. Liu *et al.* [84] through the observation of thermal effects, such as melting and solidification in Ag nanowires, could precisely identify the heating location in the structures. A similar approach has been also applied to obtain plasmonic welding in silver nanowire junctions through a self limiting process that ends the melting when the connection between the wires is formed [85]. While melting and pre-melting behaviors are very important to thoroughly understand the heating effects on plasmonic structures, sometimes even a relative small increase in temperature can be very important. For instance, regarding medical applications, a 15–20 K increase of temperature can be sufficient to kill tumoral cells inducing apoptosis [5]. Therefore, the use of metallic NP can play a terrific role in cancer treatment. In fact, nano-sized metallic structures enable a precise localization of the heating in very narrow areas and thus allowing very specific targeting. Moreover, the field resonance plays another fundamental role allowing the use of lower incident power, hence preventing heating damages in healthy nearby regions. Other applications such as smart delivery has been also investigated [5]. Here, studies regarding the LSPRs tunability are very important because at resonance the stability of the nanoparticle-molecule bond is reduced and anti-cancer drugs can be locally released. Therefore, the possibility to design ad hoc nanostructures can lead to a wide range of applications, helping in choosing the more appropriate geometries and addressing the fabrication requirements.

## 4. Influence of Temperature on Media Permittivity: The Damping Factor

In Section 2, we showed how the Drude model can be refined to include interband transitions. A permittivity based on multiple oscillators has been proved to match the experimental data fairly well, it being able to detect both the resonances positions and the absorbing behavior of metallic nanostructures. On the other hand, by simply increasing the real part of the dielectric function it is possible to find the actual resonant frequencies of any metallic nanostructure, but this procedure largely underestimates the damping factor, leading to inflated field enhancements and imprecise predictions about scattering and absorption values. Therefore, it is mandatory to deeply examine the physical origin of the damping factor, how it can be described and its dependence on the temperature.

It was mentioned that Γ describes the electrons scattering processes. In the ideal case of Γ = 0 the electrons, if already drifting, keep moving in a straight line without any interaction with the medium. As previously explained, while this condition leads to a perfect conductor in DC regime, the result is a perfect scatterer in AC regime at any frequency. In fact, even though Γ = 0 prevents the electrons from dissipating energy into heat, they can radiate while accelerating and decelerating under the influence of an external EM field [45]. This is due to the mathematical shape of *σ*(*ω*) which has been obtained through a damped Lorentzian oscillator model (see Equation (1)). Considering the motion of electrons in the conduction band of a metallic NP (but this is true for any metallic nanostructure) with Γ ≠ 0, we can imagine different kinds of scattering mechanisms such as electrons scattering with the lattice phonons (Γ*_e-ph_*), with the NP surface (Γ*_surf_*) or one another (Γ*_e-e_*). Defects are also involved in the scattering process but, in first approximation, we can neglect their contribution to the damping if sufficiently pure materials are employed. However, an approach to keep into account electrons-defects scattering will be subsequently introduced (see Equation (18)). If Γ*_e-ph_*, Γ*_surf_* and Γ*_e-e_* are the involved players, we can define a specific damping factor for each of these processes which, together, contribute to the total scattering rate:

(12) $$\begin{array}{l} \Gamma =\Gamma_{bulk}+\Gamma_{surf}\\ \Gamma_{bulk}=\Gamma_{e-e}+\Gamma_{e-ph} \end{array}$$

Here we split the bulk and the surface contributions, the latter accounting only for the electrons hitting the interface between the NP and the surrounding medium. As previously anticipated, Γ is directly linked to the plasmonic resonance lifetime τ through Γ = 1/τ, in fact the resonance survives until its associated energy is completely dissipated. To be noticed that here we do not include the radiative power originated by the plasmonic oscillations. Even though this quantity can be analytically evaluated as done for example by Grigorchuk [86], this energy “leakage” is associated with the far-field radiation emitted by accelerating and decelerating charged particles and it is thus included in the electrodynamics described by the Maxwell’s equations. This is why we have a non-zero FWHM (Full Width at Half Maximum) for a metallic NP resonance plot even if we model its permittivity by setting Γ = 0, *i.e.*, considering only the real part of the ε(*ω*) expression. The term responsible for the radiation is related to the Abraham-Lorentz force and it enables a channel through which energy can escape from the system as radiated power [45]. In 2011 Kats and coworkers [87] explicitly added this term in the complete damped harmonic oscillator equation:

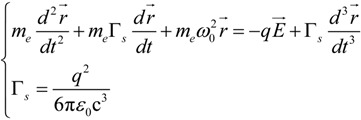
(13)


In this case, the steady state solution can be obtained as:

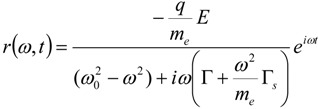
(14)


Thus we see that the total line width of the resonance is influenced by two factors: a radiative contribution proportional to Γ*_s_*, which is temperature independent, and a dissipating one which accounts for the electronic relaxation processes, Γ. To be noticed that the radiative term, due to its dependence on *ω*^2^, explains why far field scattering spectrum is blue-shifted with respect to the near field one as shown in [87].

In the next section, we will briefly examine Γ and the physical processes originating the respective damping factors.

### 4.1. Electron-Electron Scattering: Γ_e-e_

The electron-electron scattering rate was studied, for example, in 1958 by Gurzhi [88,89], and it was calculated in detail later in 1973 by Lawrence and Wilkins [90,91]. They employed the Born approximation and the Thomas-Fermi screening of Coulomb interaction to describe frequency and temperature dependence of the collision process among electrons:

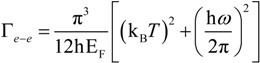
(15)


**Figure 8:**
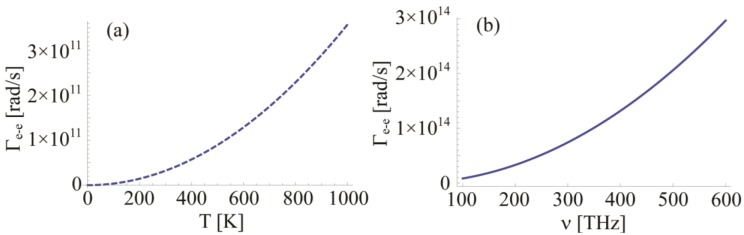
(**a**) Temperature dependence of the electron-electron scattering in DC regime (*ω* = 0); (**b**) frequency dependence of the electron-electron scattering at room temperature (*T* = 293 K). Au is considered.

When Au data are considered, Φ = 0.55 is the Fermi-surface average of scattering probability, Δ = 0.77 is the fractional Umklapp scattering coefficient, h is the Planck’s constant, *k*_B_ is the Boltzmann’s constant, *E*_F_ = 5.5 eV is the Fermi energy and *T* is the temperature. We notice that in the IR and optical frequency range the temperature dependent term turns to be negligible. However, the temperature plays a fundamental role when this scattering factor is employed to measure the DC conductivity at room temperature, as shown in Figure 8. We see that in the IR and optical range the impact of frequency on the damping value is quite important. Regarding the temperature, we understand that its contribute is clearly negligible being three orders of magnitude smaller than its frequency dependent counterpart in the optical range.

### 4.2. Electron-Phonon Scattering: Γ_e-ph_

**Figure 9:**
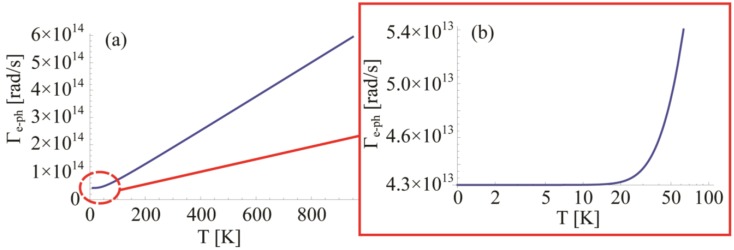
(**a**) Electron-phonon scattering dependence on temperature up to 1000 K and (**b**) zoom at low temperature. Au is considered.

While for the electron-electron scattering the temperature plays a minor role in the optical range, the electron-phonon scattering is sensitively dependent on the environment condition. Holstein [92,93] derived its expression assuming free electrons without Umklapp collisions and a single Debye model phonon spectrum:

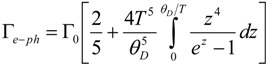
(16)
where, for gold, *θ_D_* = 170 K is the Debye’s temperature, Γ_0_ = 0.07 eV has been obtained by fitting Au bulk permittivity at frequency below 2.4 eV which is the gold interband transition onset [54]. However, this parameter can be extrapolated also from the DC resistivity in the case of isotropic scattering [94,95]; nevertheless, this latter method would underestimate Γ_0_ if the Fermi velocity was assumed position dependent on the Fermi surface [95]. Moreover, as explained in [95], the phonon behavior can be more complicated than the one assumed by Holstein (*i*.*e*., Debye dispersion), in fact for *T* *→* 0 the 2/5 factor has to be modified in the infrared phonon-assisted expression. The values for Γ*_e-ph_* in a wide range of temperatures are plotted in Figure 9. We see that from 293 K (room temperature) to 750 K (at which pre-melting has been observed [96]) Γ*_e-ph_* increases about three times. At very low temperatures, Γ*_e-ph_* begins to saturate and from 100 K we can see an almost linear behavior. The Debye temperature *θ_D_* can be seen as the temperature above which all the modes are excited and can participate to the scattering process. Interestingly, this information is easily shown by the curve shape in case of the specific heat [43], however in Γ*_e-ph_* it cannot be directly associated with the plot trend.

### 4.3. Electron-Surface Scattering: Γ_surf_

**Figure 10:**
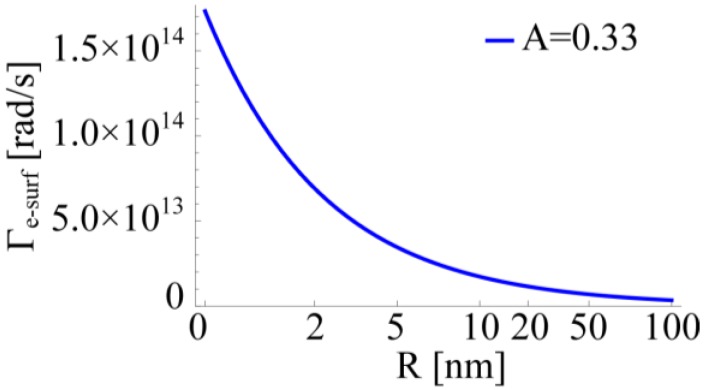
Electron-surface scattering for a gold sphere with different radii. A shape factor *A* = 0.33 has been chosen.

A standard approach to describe the damping effect due to the limited size of a nanostructure is to consider the scattering determined by the particle surface on the conduction electrons [97]. This leads to a term including a reduced mean free path of electrons when the effective length of the system shrinks:

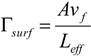
(17)
where *A* is a dimensionless parameter which takes into account the details of the scattering mechanism, *v_f_* = 1.4 × 10^6^ m/s is the gold Fermi velocity and *L_eff_* is the reduced mean free path of electrons. It is clear that the latter term is crucial since it carries all the information about the geometry of the system. In 2003 Coronado and Schatz [98] followed a geometrical probability approach to predict a general form for *L_eff_*:

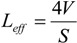
(18)
where *V* and *S* are the volume and surface area of an arbitrary shaped convex particle. This expression assumes totally inelastic scattering from the surface and it expresses the average chord length by any two points of the surface [99]. The value of *A* is still debated and empirically depends on the shape of the considered particles. Coronado in [98] mentions that its value is closed to unity, but in 2005 Berciaud *et al.* [51] found *A* = 0.33 as the best fitting value for individual gold nanoparticles absorption measurements. This value has been validated also for nanorods in 2006 by Novo and coworkers [100] who confirmed the results obtained in [51]. However, different materials may present different values of *A*: in 2004 Liu and Guyot-Sionnest [99] found for Au/Ag core-shell nanorods different values for metal/metal (*A* *~* 0.1) and metal/external medium (*A* *~* 0.25) interfaces suggesting a more inelastic scattering at the metallic interface. In Figure 10 we plot the radius dependence of electron-surface scattering for a gold nanosphere using *A* = 0.33.

We see that below 10 nm, the influence becomes quite important, and the values are comparable with electron-electron and electron-phonon scattering factors in the optical range at room temperature.

Furthermore, for very small NPs, other phenomena may occur in modifying the electric permittivity of metals. In 2001, Cai and co-workers [101] highlighted the importance of lattice contraction in shifting the surface plasmon resonances of silver NPs with dimensions between 2 nm and 10 nm. This effect is related to the change of the lattice constant of small particles induced by surface or interface stress, depending on the hosting matrix. For a cubic lattice and a spherical structure, lattice contraction due to surface stress *f* (or interface stress for embedded NPs) can be calculated as [102]:

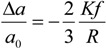
(19)


Here *K* is the compressibility and *R* the particle radius. As explained in [101], variations of lattice constants modify the electron density thus shifting the Mie resonances. This phenomenon adds up to the free path effect previously mentioned. However, whereas a reduced free path implies a red-shift of the Mie frequency, lattice contraction pushes the resonance towards higher frequencies as long as *f* is positive. Therefore, the effects can partially counterbalance each other. In fact, generally lattice parameters are expected to diminish with decreasing size; nevertheless lattice dilatation and negative stress are reported in literature [103].

The Mie resonance shifting due to lattice contraction can also be exploited to obtain photoelastic coefficient in noble metals as suggested by Temnov [104]. However, within this review, and especially in the following simulations, we consider bigger NPs (*R =* 10 nm), therefore we will neglect this effect in our modeling of the electric permittivity of gold.

## 5. A Temperature Dependent Permittivity Model with Interband Transitions

Now that we have introduced the main quantities affecting the electric permittivity, we can merge them together to define a *temperature dependent non-linear permittivity*. Its expression will be formed by two terms: the first one taking into account the conduction electrons and the influence of temperature, the second one expressing the interband absorptions:

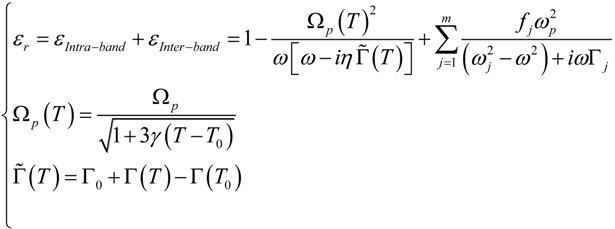
(20)
where Γ(*T*) = Γ*_e-e_*(*T*) + Γ*_e-ph_*(*T*) + Γ*_e-surf_* is the total damping coefficient at temperature T. When gold is considered, *γ* = 14.2 × 10^−6^ K^−1^ is the thermal expansion coefficient, Γ_0_ = 8.04 × 10^13^ rad/s is the intraband damping coefficient [50], *η* is a dimensionless parameter which can be tuned to take into account defects-induced damping changes [105] and *T*_0_ = 293.15 K is the room temperature. In our simulations we will set *η =* 1, therefore neglecting damping adjustments due to the roughness or defects. We started by considering the Drude-Lorentz model where the intraband part was modified by inserting the temperature dependence of the plasma frequency, that includes a reduced electronic density due to the thermal volume expansion [54]. Furthermore, to preserve the Drude-Lorentz model behavior at room temperature, we introduced the damping parameters in the model by subtracting their contributes calculated at *T*_0_. In this way, at room temperature, our model can exactly reproduce the Drude-Lorentz model but, at the same time, we could include the dependence from the temperature. For simplicity, we will refer to this model as DLT (Drude-Lorentz-Temperature model).

By using DLT we can numerically calculate the impact of the temperature on plasmonic nanostructures. In particular, we will show how resonances, field enhancement and scattering parameters are going to change with the temperature through a non linear iterative calculation implemented in a FEM (Finite Element Method) numerical technique.

In the following simulations electromagnetic equations have been solved, through iterative calculations, along with the heat diffusion equation:

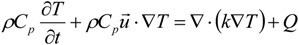
(21)


Here *ρ* represents the material density, *C_p_* the thermal capacity at constant pressure, u the velocity vector, *k* the thermal conductivity and *Q* the amount of heat provided to the system. In this case, *Q* is associated with the electromagnetic losses related to the Joule effect, as previously explained.

In the hypothesis of stationary regime, this equation can be easily simplified. In particular, by considering that only the conductive heat transfer term is feasible (*i*.*e*., the convection term is by definition zero inside the solid structure and it has been neglected in the surrounding medium), the equation solved for temperature *T* becomes:

− ∇ · (*k*∇*T*) = *Q* (22)

### 5.1. Tuning the Overall Temperature

**Figure 11:**
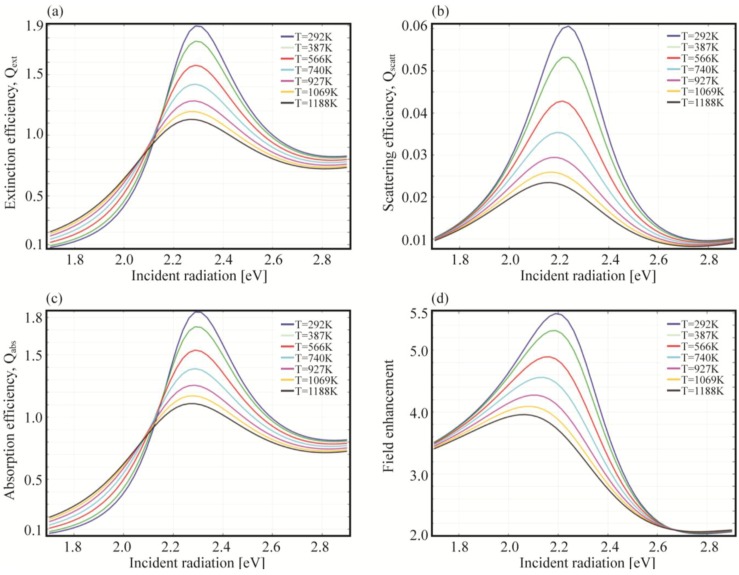
(**a**) Extinction efficiency; (**b**) scattering efficiency; (**c**) absorption efficiency; and (**d**) field enhancement for a gold nanospheres (*R* = 10 nm) embedded in silica at different temperatures.

The first simulation considers a spherical nanoparticle with radius *R* = 10 nm embedded in a fused silica host matrix in interaction with a low power Gaussian beam (beam size *w*_0_ = 1 μm). We choose a low power source in such a way that the temperature rise, due to the dissipated power, can be neglected. In the simulations, in order to modify the values of the damping parameters, we tuned the overall temperature. The chosen *T*_0_ values will be the same as reported in [96]. In Figure 11, the plots showing extinction, absorption, scattering efficiency and electric field enhancement at 1 nm over the NP surface are reported.

In the figures, we can immediately observe how the increase of temperature lowers the value of all the selected quantities, while broadening the spectra across the resonance. It is interesting to notice how the scattering efficiency and the near field enhancement peaks shift with the temperature, while the absorption seems to depend on it only marginally. This can be understood by means of the mechanisms ruling the different optimal condition for maximizing absorption or scattering. Later on (see Section 5.2), when we will tune the incident power, thus triggering the system non linearities, we will clarify the origin of this shift.

Another shift can be observed between the near and far field (scattering efficiency) peaks, at the same temperature. This can be explained in terms of radiation damping Γ_rad_ as previously anticipated [87] (see Section 4). Furthermore, by comparing the absorption and scattering efficiency, we notice the former to be at least one order of magnitude higher than the latter. The explanation is in the dimensions of the particle, in fact the radiation damping is proportional to the particle volume, namely the bigger the particle the higher the scattering efficiency [73]. This is illustrated by the following set of equations which relates absorption and scattering efficiencies, radiative and non radiative damping and polarizability:

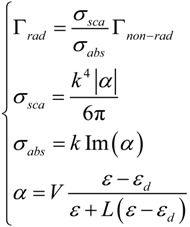
(23)
where *α* is the particle polarizability written in a more generalized form as reported in [106], *k* the vacuum wave vector of the incident radiation, *ε* the dispersive dielectric function, *ε_d_* the surrounding medium permittivity, *V* the particle volume and *L* the depolarization factor which accounts for the particle shape and orientation. To be noted that here we explicitly distinguished the damping due to radiated energy (Γ*_rad_*) from the damping associated with dissipated energy into heat (Γ*_non-rad_*). If retardation effects are neglected and if *ε* is described by means of a simple lossy Drude model (see Equation (4)), the radiative damping can be expressed as [106]:

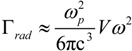
(24)


This formula highlights the strong dependence of the damping on both *ω* and the particle volume *V* and it can be associated to the Abraham-Lorentz term mentioned before in Equation (12).

### 5.2. Tuning the Incident Power

**Figure 12:**
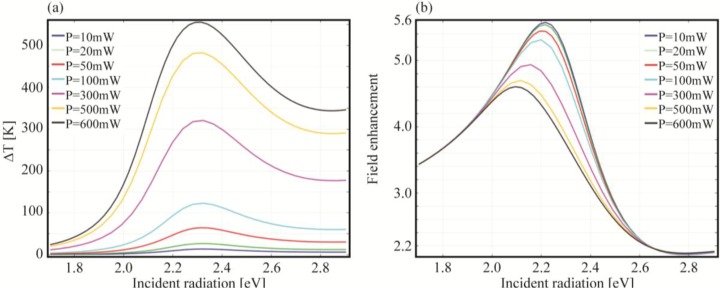
(**a**) Calculated temperature increase; and (**b**) field enhancement spectra at 1 nm over the surface for a gold nanosphere (*R* = 10 nm) embedded in silica at different incident powers.). The incident field has been modeled as a Gaussian wave with beam size *w*_0_ = 1 m.

In the previous calculations, we homogeneously raised the whole system temperature hence inducing an increase in the damping factor values. Another possibility is to keep the system at room temperature, instead hitting the sample with a focused laser carrying higher power. In this way, we can determine how the resonances influence the temperature around the NP and how non linearities are triggered in the system. In fact, the incident power at different wavelengths interacts with the sample according to its permittivity values. This implies different heating efficiencies, which lead to different temperatures, which, in turn, modify the damping factors and thus the dielectric function. Therefore, differently from the previous simulations, here the system enters in a non linear loop thus making the NP refractive index directly dependent on both frequency and intensity *n*(*ω*,*I*). In Figure 12, we report the field enhancement and the temperature increase reached at 1 nm outside a gold nanosphere for different incident powers, ranging from 10 mW to 600 mW. In analogy with previous calculations, the NP is placed in a silica host matrix with thermal conductivity *κ_SiO_2__* = 1.4 [W/(m·K)], heat capacity *c_p_*,*_SiO_2__* = 730 [J/(Kg·K)] and density *ρ_SiO_2__* = 2.2 × 10^3^ [Kg/m^3^]. The utilized thermal data for gold are *κ_Au_* = 317 [W/(m·K)], *c_p,Au_* = 129 [J/(Kg·K)] and *ρ_Au_* = 1.93 × 10^4^ [Kg/m^3^] [107].

**Figure 13:**
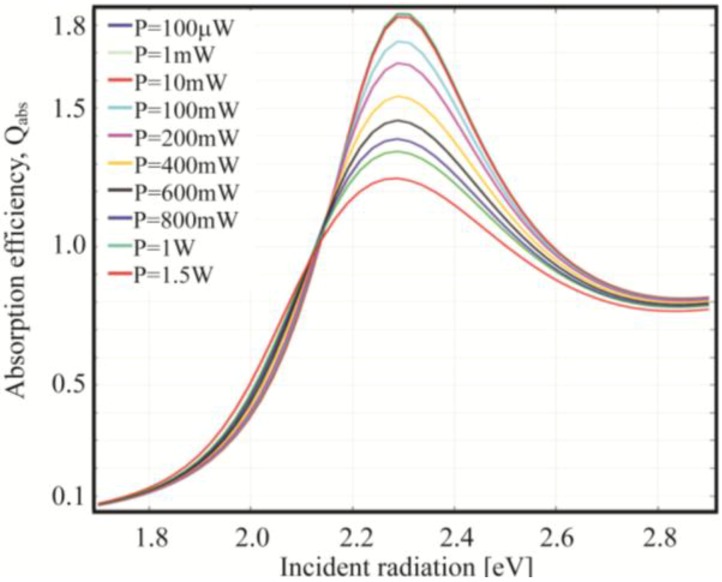
Calculated absorption efficiency spectra for a *R* = 10 nm gold nanosphere embedded in silica for growing input powers.

Interestingly, in Figure 12 we observe, for a given incident power, a wide range of Δ*T* = *T* *−* *T*_0_ depending on the chosen frequency. For instance, taking the maximum power, 600 mW, we see that the temperature rises by roughly 20 K at 1.7 eV while jumps to almost 550 K at 2.3 eV. In fact, far from the field enhancement resonance, the curves overlap for any considered power: the temperature increase is minimal and thus damping parameters do not change leading to similar results even though the input power is tuned. The situation is different around resonance where the temperature increases sensitively modifying the damping parameters thus changing the behavior of the NP. In the previous section the temperature was uniformly changed all over the considered spectrum while now we see that it strongly depends on the optical response of the nanoparticle. In Figure 13 is plotted the absorption efficiency in the range from 100 W to 1.5 W.

**Figure 14:**
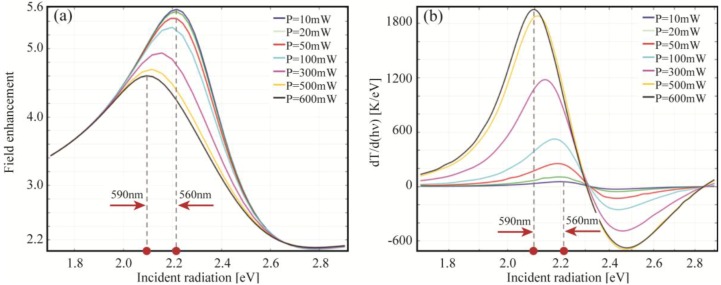
(**a**) Field enhancement; and (**b**) temperature derivative with respect to incident radiation energy for a gold nanospheres (*R* = 10 nm) embedded in silica at different incident powers.

Another interesting relation, always standing for any incident power, can be found between the electric field resonances and the temperature derivative with respect to the incident radiation energy. In Figure 14 the field enhancement is plotted next to the derivative of the temperature (both measured at 1 nm from the NP).

**Figure 15:**
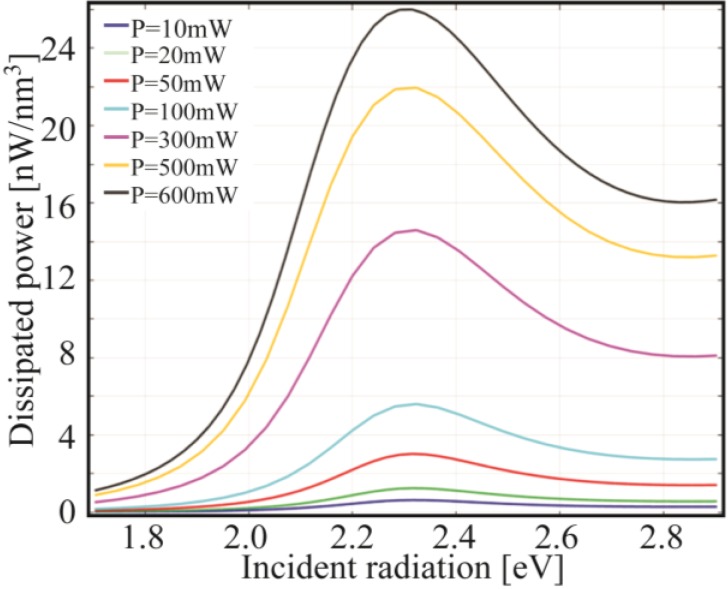
Dissipated power (Joule heating) spectra for a gold nanospheres (*R* = 10 nm) embedded in Silica at different incident powers.

It is important to notice that the two graphs are each other strongly connected inasmuch as the electric field maxima correspond to the maxima of the temperature derivative. In fact, the temperature derivative with respect to the incident energy tells us how much the temperature is likely to change in a certain energy range. From 1.7 eV to 2.3 eV the derivative is positive, thus the temperature rises towards its maximum value which, in fact, is reached at 2.3 eV, as it can be viewed in Figure 14. At 2.3 eV the NP is indeed converting the maximum portion of incident energy into heat, as confirmed by the absorption efficiency plot (see Figure 13). The peaks showed by the derivative between 1.7 eV and 2.3 eV can be explained in terms of dissipated power due to the Joule heating. In fact, we can observe that the spectra of the dissipated power (Figure 15) have the same shape of the temperature increase (Figure 12).

**Figure 16:**
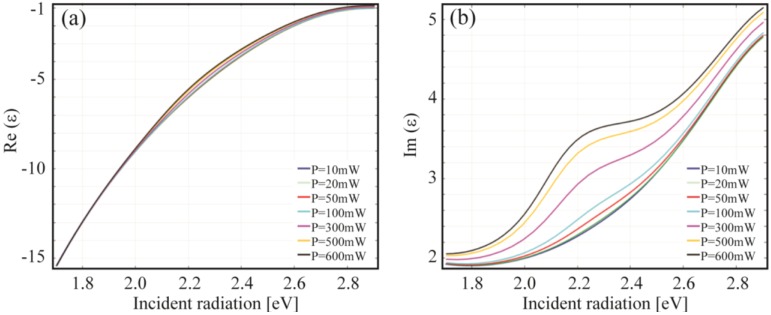
Power dependence of (**a**) the real and (**b**) imaginary part of the dielectric function (Equation (18)) for a gold nanosphere (*R* = 10 nm) embedded in Silica.

In Section 2 was mentioned that the electric field resonant condition is given by the proper match between the real part of the permittivity and the nanostructure geometry. However, when temperature effects (Γ(*T*)) are considered, we might expect some influence on the resonance position (see Equation (18) and Figure 14). Interestingly, as shown in Figure 16, this effect is only weakly related to Re(*ε*), instead it is the imaginary part of the permittivity that undergoes a sensitive modification due to temperature (power) change.

Similar considerations on the marginal role of the real part of the permittivity can be found in [40] where optical effects where discussed at low temperature.

In order to understand the shift of the near field enhancement shown in Figure 14, different considerations have then to be taken into account. From Figure 15 we clearly see how the steepness of the curves changes depending on the intensity values. In particular, in the same incident radiation energy range (from 1.8 eV to 2.2 eV), the amount of power dissipated grows faster at higher intensity. Therefore, the amount of power available for scattering (*i.e.*, the part which is not dissipated) tends to increase for lower energy values, which is especially true for high intensities (e.g., the steepness at 600 mW is much higher than at 50 mW). This mechanism is behind the red-shift behavior observed in the near-field enhancement shown in Figure 14.

## 6. Final Considerations

We have seen that in order to numerically reproduce more realistic systems, where temperature related phenomena are an issue, it was necessary to properly improve the standard modeling of the permittivity. Temperature effects, together with dispersion relations, on host matrix should be considered since high temperature can indeed modify their permittivities [108]. When very high temperatures are taken into account, also the thermal expansion could play a role in plasmonic resonances. However the influence that this quantity might have on the electric permittivity is still debated: in [54] it is found that for an array Au rods the expansion is below 1.6 × 10^−2^ nm/K and thus small compared to permittivity temperature dependence. On the other hand, in [96], the thermal expansion was inserted in the permittivity model to explain absorption shifts in gold nanoparticles heated at different temperatures. Furthermore, other promising applications can derive from the coupling between heating and convective phenomena when particles are immersed in liquid environments. In 2011 Donner *et al.* [109] showed that fluid velocity due to plasmonic heating can be neglected for single nano-sized particles but it is relevant when considering micro-sized systems or NPs ensembles. New modeling challenges come also from the emerging theme of superconducting plasmonics. About this subject, recently Tsiatmas and co-workers [110] proposed a generalized Drude model where a lossless term, representing the contribution of non-dissipating Cooper pairs, was added to the lossymetallic dispersion.

Another emerging field linking nanophotonics to the plasmonic heating can be found in ultrafast optics where non-equilibrium electrons are created and non-linear effects are triggered [104,111,112,113,114]. In fact, when an ultrashort pulse is absorbed by free carriers in a metal, a non-equilibrium distribution of electrons is created within the skin depth of the medium [104]. Electrons then thermalize through electron-electron scattering in a time frame between tens to hundreds of femtoseconds. For silver and gold, relaxation times of 350 fs and 500 fs have been found respectively [115]. However, even after electronic thermalization, the system still remains in non-equilibrium since electronic temperature is much higher than the lattice one. At this point the electron-phonon interaction cools down the hot electrons and the lattice heats up reaching equilibrium conditions in the order of picoseconds [75,104]. Within this context, ultrafast acousto- or magneto-plasmonics offer even more challenges in terms of modeling perspectives. In these cases, spatial dependent strain and magnetization can modify the electric permittivity of metals and, in principle, the latter can be engineered to obtain peculiar and more complex optical responses [104].

All these topics give an idea of how important is a proper description of the media optical response. Moreover, it is interesting to realize how a relatively simple model based on several assumptions elaborated at the beginning of 1900 can be continuously improved to describe new optical phenomena.

## 7. Conclusions

In this review, we described how a plasmonic system can be optically and thermally modeled. We analyzed the relationships among macroscopic media parameters, such as refractive index, electric conductivity and dielectric permittivity showing how they can be used to fully describe the dispersion behavior of a metallic nanostructure. We investigated the connection between dielectric function and the heating properties of resonant plasmonic NPs. We expanded the damping factor in a number of terms, each one of them related to a microscopic scattering phenomenon. Finally, we merged all these terms together in order to define a dielectric function capable of predicting the plasmonic behavior at different temperature regimes. The numerical calculations were realized by implementing in a FEM architecture a non-linear calculation regime which could account for the incidence intensity dependence on the refractive index of the metallic structures.

We have found that any temperature change can strongly modify the field enhancement and absorption characteristics of plasmonic devices. The imaginary part of the permittivity has been shown to double (at around 800 K) its value with respect to the room temperature at resonant wavelength. Interestingly, for a given incidence power, a very different temperature increase can be induced in the NP. Owing to our non-linear calculation method, it was shown that for a gold NP the temperature rise might fall in the range from 30 K to 500 K depending on the chosen incident power and frequency. This result expresses a very high sensitivity of the absorption properties of the system. Another important finding is that the optical and thermal hot spots do not necessary coincide. In fact, scattering and absorption are indeed two parallel energy leaking channels acting at the same time.

Finally, a deep understanding of the optical and thermal phenomena in plasmonic structures has a great importance in the study of metallic systems. Improved modeling techniques and advanced calculation possibilities can thus be tremendously useful for both new technological applications and for a better understanding of the microscopic electronic behavior in nano-sized structures.
